# p21^−/−^ mice exhibit enhanced bone regeneration after injury

**DOI:** 10.1186/s12891-017-1790-z

**Published:** 2017-11-09

**Authors:** Priyatha Premnath, Britta Jorgenson, Ricarda Hess, Pankaj Tailor, Dante Louie, Jaymi Taiani, Steven Boyd, Roman Krawetz

**Affiliations:** 1 McCaig Institute for Bone and Joint Health, Cumming School of Medicine, University of Calgary, Calgary, AB Canada; 2 Department of Cell Biology and Anatomy, Cumming School of Medicine, University of Calgary, Calgary, AB Canada; 3 Biomedical Engineering Graduate Program, University of Calgary, Calgary, AB Canada; 4 Department of Surgery, Cumming School of Medicine, University of Calgary, Calgary, AB Canada; 5 Department of Radiology, Cumming School of Medicine, University of Calgary, Calgary, AB Canada; 6 Snyder Institute, Cummings School of Medicine, University of Calgary, HRIC 3AA14, 3330 Hospital Dr. NW., Calgary, AB T2N 4N1 Canada

**Keywords:** p21, Bone healing, Trabecular bone, μCT, Mice

## Abstract

**Background:**

p21^(WAF1/CIP1/SDI1)^, a cyclin dependent kinase inhibitor has been shown to influence cell proliferation, differentiation and apoptosis; but more recently, p21 has been implicated in tissue repair. Studies on p21(^−/−^) knockout mice have demonstrated results that vary from complete regeneration and healing of tissue to attenuated healing. There have however been no studies that have evaluated the role of p21 inhibition in bone healing and remodeling.

**Methods:**

The current study employs a burr-hole fracture model to investigate bone regeneration subsequent to an injury in a p21^−/−^ mouse model. p21^−/−^ and C57BL/6 mice were subjected to a burr-hole fracture on their proximal tibia, and their bony parameters were measured over 4 weeks via in vivo μCT scanning.

**Results:**

p21^−/−^ mice present with enhanced healing from week 1 through week 4. Differences in bone formation and resorption potential between the two mouse models are assessed via quantitative and functional assays. While the μCT analysis indicates that p21^−/−^ mice have enhanced bone healing capabilities, it appears that the differences observed may not be due to the function of osteoblasts or osteoclasts. Furthermore, no differences were observed in the differentiation of progenitor cells (mesenchymal or monocytic) into osteoblasts or osteoclasts respectively.

**Conclusions:**

Therefore, it remains unknown how p21 is regulating enhanced fracture repair and further studies are required to determine which cell type(s) are responsible for this regenerative phenotype.

**Electronic supplementary material:**

The online version of this article (10.1186/s12891-017-1790-z) contains supplementary material, which is available to authorized users.

## Background

The natural remodeling process of the bone is effective in maintaining tissue homeostasis, however, a diversity of medical conditions exist that adversely affect the mechanical and biological integrity of the tissue such as physical injuries to the bone, osteoporosis, cancer and inflammatory bone disease [1]. Current clinical interventions include immobilization with metal rods and pins, implantation of autologous or allogeneic bone grafts and implantation of metals or ceramics. Effective immobilization can be challenging to achieve due to the deteriorated state of the bone [2, 3]. Although treatment using bone grafts is quite effective, this method is accompanied by serious limitations, including limited supply, donor site morbidity and, in the case of allografts, risk of rejection or disease transmission [4]. The use of metals or ceramic implants have clear disadvantages in that they exhibit limited integration with the host tissue and can fail as a result of infection [4]. Gene therapies have been proposed as viable options, both in in vivo and ex vivo models [5]. Gene therapies provide methods to stimulate molecules or groups of molecules to enhance bone regeneration [6]. For example, the inhibition of p21 has been observed to increase cell proliferation and subsequently cause regeneration of tissues [7, 8]. p21 ^WAF1/CIP1/SDI1^, a cyclin dependent kinase inhibitor, is intricately involved in cell proliferation, acting at the G_1_ check point during cell cycle. In essence, p21 inhibits the activity of cyclin/cdk2 complexes and inversely affects cell cycle progression [9].

To evaluate the role of proliferation in tissue repair, a previous study examined fibroblasts obtained from ‘healer’ and ‘non-healer’ mice and tested for their regenerative properties based on a G_1_ cell cycle checkpoint deficiency. It was observed that deletion of p21 alone was sufficient to convert non-healer mice into appendage regeneration-competent healers: ear hole closure in 6–7 week old p21^−/−^ mice was only slightly less than that of MRL (Murphy Roths Large, super healer) mice by 28 days after injury [7]. In another study, the role of p21 inhibition in liver regeneration was probed, and, continuous hepatocyte proliferation was observed, albeit while also facilitating tumor development [8]. There have been no studies on specifically cartilage and bone regeneration in p21^−/−^ mice. Nonetheless, there have been studies on skeletal muscle regeneration and embryonic endochondral ossification, where interestingly enhanced repair was not obtained; specifically, p21^−/−^ mice display delayed regeneration of muscle after injury compared to wildtype controls [10]. While several studies assessed p21 inhibition in adult mouse tissue, Chinzei et al. evaluated the role of p21 in embryonic endochondral ossification in vivo, and found that p21 deficiency appears not to influence ossification with other signaling pathways likely compensating [11]. Despite the lack of studies on the role of p21 in bone and cartilage, other members of the p21 signaling pathway, such as E2F1 have been investigated and could potentially predict the role of p21within bone development/repair. E2F1 like p21 is also involved intricately in proliferation and apoptosis, where its transactivation is governed by pRb (retinoblastoma protein) [12]. E2F1 and p21 are inversely related, inhibition of E2F1 leads to upregulation of p21, thereby decreasing cell proliferation [13]. As previously described, the Chinzei et al. study established that p21 inhibition did not have an effect on endochondral ossification, however, Scheijen et al., demonstrated that overexpression of E2F1 delayed endochondral bone formation owing to altered chondrocyte maturation [14]. In contrast, when E2F1 was inhibited in a Rb-deficient embryo, bone defects caused by Rb deficiency was reversed, demonstrating a role of E2F1 in osteoblast differentiation [15]. These results, taken together suggest that the p21-E2F1 signaling pathway may play a role in bone development and/or repair after injury, however, to date no study has examined if p21 KO mice demonstrate enhanced fracture repair.

The process of bone healing proceeds with the recruitment and differentiation of mesenchymal stem cells into skeletal and vascular tissues. A cartilaginous callus is formed which is followed by mineralization of the extra cellular matrix. While cartilage is resorbed, bone formation is initiated, and bone remodeling commences where osteoclasts resorb primary bone and secondary bone is formed [16]. Differentiation of mesenchymal stem cells into osteoblasts is an important factor in determining bone healing and remodeling. The knockdown of p21 in late-passage mesenchymal stem cells (MSCs) exhibited increased proliferation capacity and increased osteogenic potential [17]. It has been previously shown that there are inherent differences in p21 expression in mesenchymal progenitor cells between normal and osteoarthritic patients; with increased p21 levels correlated with decreased chondrogenic potential [18].

These studies provide a strong rationale to examine the role of p21 in bone repair; with other kinase inhibitors having being examined in this area of research previously. Drissi et al., demonstrated that osteo-progenitor cells derived from bone marrow of p27^−/−^ mice proliferated at increased rates compared to wild type mice while retaining differentiation capacity; they also suggested that increased p21 levels were linked to retained differentiation capacity [19]. These results indicate that kinase inhibitors may play an important role in bone remodeling. While p21 has been implicated in bone remodeling through p27^−/−^ mice, it has been previously observed that deletion of p21 did not alter embryonic endochondral ossification in mice, as stated earlier [20]. There have been no studies that have directly examined the role of p21 in bone regeneration, specifically after an injury. Therefore, this study was designed to examine the role of p21 in a non-critical size fracture model [21].

## Methods

### Animal model

p21^−/−^ mice (B6;129S2-Cdkn1atm1Tyj/J, Jackson labs) backcrossed onto a C57BL/6 background and wild-type C57BL/6 mice (Jackson labs) were used in this study. Twelve 8 week old mice were used in each group that included: p21^−/−^ mice with injury (*n* = 12), without injury (radiation control) (*n* = 12), and C57BL/6 mice with injury (*n* = 12). Our previous studies have already generated microarchitectural measurements for C57BL/6 mice without injury (radiation controls) [21]. The injury groups had a burr-hole fracture on the right limb and the left limb had no injury.

### Burr-hole fracture model

All animal procedures are approved by the University of Calgary Animal Care Committee. The burr-hole model fracture was performed based on a protocol described by Taiani et al. [22]. Prior to surgery, the mice were anaesthetized using isoflurane followed by a subcutaneous administration of Buprenophine (0.05 mg/kg) and Derapen™(1 mL/15 kg). The lower torso of the animal was sterilized with alcohol and then a small incision was made on the medial side of the right knee. A high-speed microdrill (Fine Science Tools, Vancouver, BC, Canada) was used to drill through the medial cortex and through the medullary cavity of the metaphysis, using a 0.7 mm burr. The skin was closed using a cyanoacrylate (Vetbond) tissue adhesive (3 M). The 12 radiation control p21^−/−^ mice were divided into two groups, 6 underwent sham surgery that included scraping of tissue from tibia, and an additional 6 mice had no surgical procedure conducted. Subsequent to the surgery, the mice were returned to their cages and allowed to bear weight immediately. At week 5, the mice were sacrificed and the dimensions of the tibiae (length, diameter) were measured, and the tibiae were collected for flow cytometry and histology/immunohistochemistry.

### In vivo micro-computed tomography (μCT)

Trabecular bone morphology and bone mineral density (BMD) was measured via in vivo μCT scanning of fractured tibia over a period of four weeks. Radiation controls for p21^−/−^ and C57BL/6 mice were scanned at the same time points but analyzed at day 0 and week 4 only. Radiation control μCT data for C57BL/6 mice was obtained from a previous study to reduce animal numbers [21].Day 0 scans were conducted immediately after the surgery to ascertain a baseline for trabecular morphology and bone mineral density. For μCT scanning, mice were anesthetized using 1.5 vol/vol% isoflurane with 1 L/min oxygen for the entire duration of the scan, which lasted approximately 20 min. The proximal 6.36 mm of the fractured right limb and uninjured left limb were scanned at an isotropic resolution of 15 μm (μCT 40, Scanco Medical AG, Basserdorf, Switzerland) at a tube voltage of 45 kVp, tube current of 133 μA, integration time of 200 ms, consisting of 1000 projections over 180^°^ and reconstructed on a 2048 X 2048 matrix. The radiation dose is 360 mGy for each scan. The scanner was calibrated using hydroxyapatite phantoms.

For analysis, the proximal and distal ends of the bone were cropped to generate a region of interest (ROI) consisting of 100 slices, or 1.5 mm, centered at the burr-hole. This approach was necessary to study bony changes exclusively at the site of the fracture. The scans from week 1, 2 and 4 are registered to their day 0 counterparts to relatively assess bony changes. After the scans were completed, cortical and trabecular bone regions were extracted using Image Processing Language (IPL V5.08b, Scanco Medical, Brüttisellen, Switzerland) via segmentation [23]. The mean gray-scale values of voxels in the cortical and trabecular region are converted to HA/cm^3^ to be reported as bone mineral density. Trabecular bone volume ratio (BV/TV), trabecular thickness (Tb.Th), trabecular separation (Tb.Sp), trabecular number (Tb.N) and trabecular connectivity density (Conn.D) were calculated from ROI after segmentation and registration. Data normalization was conducted using the average of tibia dimensions from each mouse group following a method similar to Saint et al. [24].

### Cell analysis and molecular analysis

#### Flow Cytometry

Immediately after the mice were euthanized, the fractured right tibiae were dissected and the bone marrow was flushed with a 26 Gauge needle. The resulting cells were labelled for stem cell markers, CD140a (Catalog Number: 558774BD Biosciences), Sca1 (Catalog Number: 557,405, BD Biosciences) and quantified via flow cytometry. CD140a and Sca1 were labelled according to manufacturer’s instructions. The cells were fixed and permeabilized prior to labelling.

In order to study inherent differences in osteoblast and osteoclast numbers between p21^−/−^ and C57BL/6 mice, the right femurs from the injured mice were dissected immediately after the mice were euthanized. The bone marrow was flushed out using a 26-gauge syringe (BD). The cells were then stained for osteoblasts using sp7/osterix (OSX) (Catalog Number: Sc-393,325, Santa Cruz Biotechnology)- conjugated to Alexa Flour 546, and for osteoclasts using TRAP (Tartrate-resistant acid phosphatase) (Catalog Number: sc-30,833, Santa Cruz Biotechnology) - conjugated to Alexa Flour 647. Similar to the stem cell study, bone marrow cells were fixed, permeabilized and then labelled with the antibodies. Flow cytometry was repeated 3 times to confirm results, including with female and male mice separately.

#### MSC isolation and purification

The uninjured tibiae and femur from both mice were extracted and flushed. The total isolated cell fraction was plated in a 6 well plate and allowed to adhere overnight at 37 °C and 5% CO_2_ in DMEM/F12 media. Half the media was replaced after 24 h. The cells were grown to confluence. Once the cells were grown to confluence in a T-25 flask, medium was removed and cells were trypsinized with 0.25% trypsin-EDTA (Life Technologies) until cells were seen to lift from the plate, after which medium was added to deactivate the trypsin. The cell suspension was then centrifuged for 5 min. A working solution of BD IMAG buffer (BD Biosciences) was produced by mixing 1 mL of BD IMAG Buffer (10×) with 9 mL of sterile distilled water. The solution was kept on ice for the duration of the procedure. After centrifugation, the supernatant was discarded and the cell pellet was resuspended in 1 ml of diluted BD buffer. Five micro-litres of BD immune cell depletion cocktail was added to the cell suspension, placed on ice for 15 min after which 5 uL of magnetic particles (BD) was added. The suspension was placed in a sorting magnet (BD) for 7 min after which the liquid phase was removed and the process was repeated using a Sca1 positive magnetic selection approach, the method was the same as above but using a Sca1 biotinylated antibody (EBioscience). Following this cells were grown to confluence in T-25 flask, followed by T-75 flask when necessary.

#### Osteogenic differentiation

For osteogenic differentiation, a total of 10,000 cells/well/24well plate was used. Cells were cultured in osteogenic differentiation medium at 37 °C with 5% CO_2_ and medium changes performed every 2–3 days (as needed) for 21 days. Differentiation medium consisted of MSC culture medium with 0.1 μM dexamethasone and 50 μM ascorbate-2-phosphate.

RNA was extracted via E.Z.N.A® Total RNA kit I (OMEGA). cDNA was obtained from RNA via High Capacity Reverse Transcriptase cDNA kit (Invitrogen). Levels of mRNA were analyzed using TaqMan® Universal PCR Master Mix (Applied Biosciences) using TaqMan® Gene Expression Assay primers (Applied Biosciences) for mouse RunX2, Sp7/osterix and 18S (endogenous control) on 7900HT Fast-Real-Time PCR System. Samples were run in triplicate.

#### Osteoclast resorption assay

To examine osteoclast activity, a Corning® osteo assay was employed (Corning). Once the left femur was dissected from the mice, the bone marrow was flushed and the cells were plated on to the osteo plate according to the manufactures instructions. In order to examine differentiated osteoclasts, M-CSF (macrophage colony stimulating factor) (30 ng/ml) (Catalog Number: M9170, Sigma) and RANKL (50 ng/ml) (RANK Ligand) (Catalog Number: R-0525, Sigma) was added to the media. The media was changed daily, for 7 days. At the 7-day mark, the media was aspirated, followed by treatment with 10% bleach solution. The wells were washed three times with water and subsequently treated with 1% toluidine solution to visualize the osteoclast pits. The area of the pits was measured via Image j (1.49, NIH, USA).

#### Histology

To test the osteogenic capacity, purified stem cells were grown to confluence in osteogenic medium. They were then fixed in 10% neutral buffered formalin (NBF) for an hour. The cells were then washed with distilled water and stained with alizarin red (Sigma). The cells were incubated in the dark for 45mins. Subsequently, the cells were washed twice with distilled water. Bright orange- red extracellular calcium deposits were considered a positive stain.

Standard histology was performed to evaluate local tissue morphology via Safranin-O and Fast-Green staining. 10 μm slices were dipped in reducing concentrations of ethanol and then water. Subsequently, the slides were dipped in haematoxylin (Sigma Aldrich), followed by acid alcohol, ammonia water and fast-green (Sigma Aldrich) and safranin-O (Sigma Aldrich). Finally, the slides were dipped in increasing concentrations of ethanol. The samples were imaged via Axio Scan Z.1 (Zeiss, Germany).

#### Immunohistochemistry

Groups of C57BL/6 and p21^−/−^ mice were euthanized after 4-week post injury to assess changes to the injury site. Immediately after the mice were euthanized, the fractured right tibiae were dissected and fixed in 4% neutral buffered formalin for 5 days. The fixed tibia was decalcified in CalEx (Fisher Scientific, Canada) for 10 days, with changes every two days. The decalcified samples were embedded in paraffin, sectioned and co-stained with CD140a (BD Biosciences), Sca1 (BD Biosciences) and PRX1 (Novus Biologicals) to identify MSCs, and DAPI (Sigma) to identify nucleus. The 10 μm thickness slices were deparaffanized and underwent antigen retrieval using boiling sodium citrate solution. The samples were then blocked for non-specific binding using rat serum (Sigma Aldrich). This was followed with antibody staining.

#### Statistical analysis

The μCT data for each mouse group (*n* = 12) was compared via ANOVA and MANOVA with a Scheffe and Tukey post hoc test at each time point (IBM SPSS Statistics 22.0). Flow cytometry, qPCR and osteoclast activity were analyzed via student’s t-test. Statistical tests were discussed with a bioinformatician at the University of Calgary.

## Results

### Bone μCT parameters

**Fig. 1:**
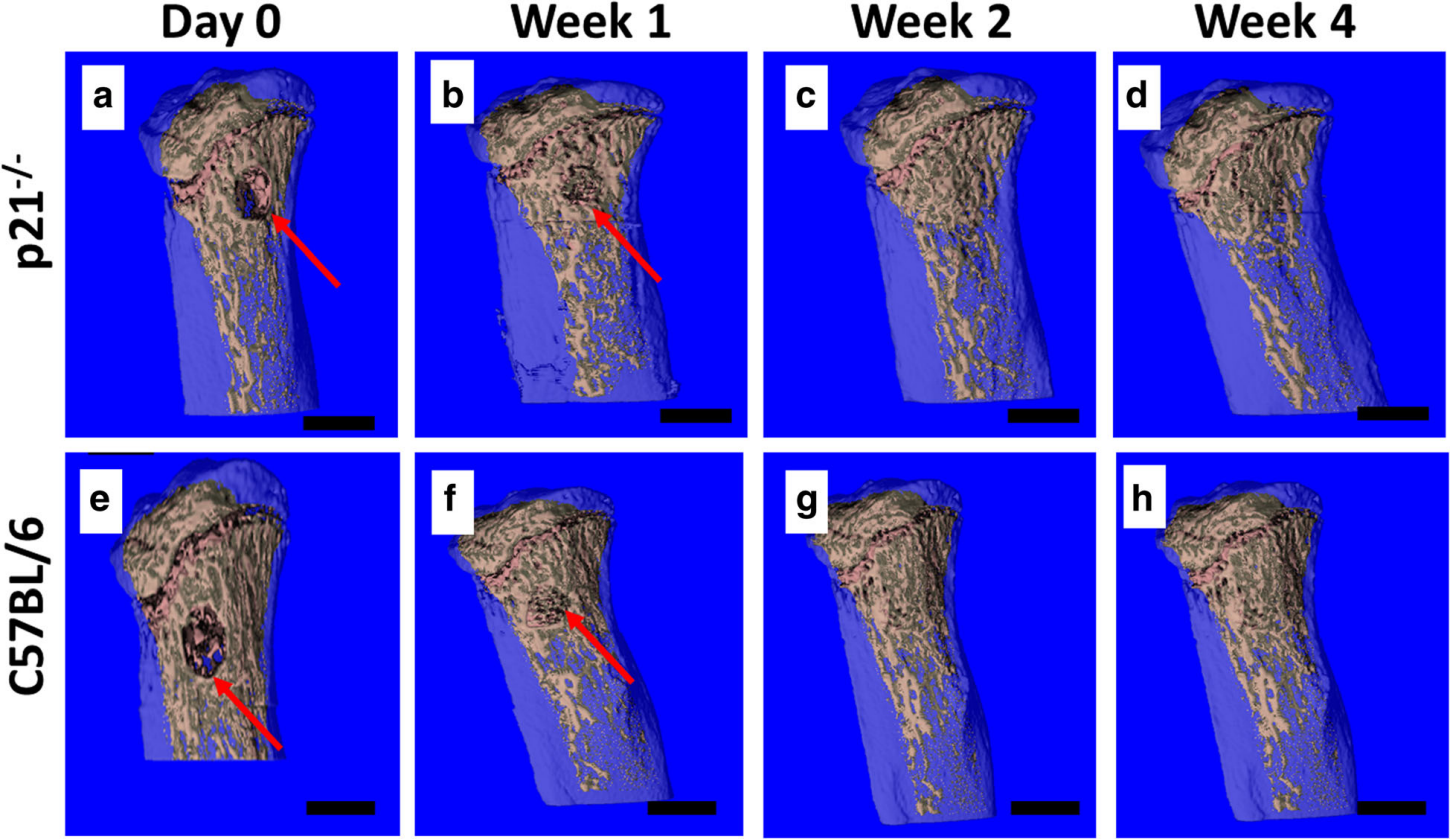
Panel of representative μCT images illustrating trabecular morphology at day 0, Week 1, 2 and 4, post burr-hole surgery of p21^−/−^ (**a**−**d**) mice and C57BL/6 (**e**−**h**) mice. It is observed that at week 1, both C57BL/6 and p21^−/−^ mice showed expected regeneration at the ROI. By week 2, the bone has returned to a pre-injury morphology and remains stable through week 4. The red arrows indicate the location of the burr-hole fracture. Each mouse group (*n*) = 12. Scale bars: 1 mm

Representative μCT scans of the tibiae of both C57BL/6 and p21^−/−^ mice immediately after surgery (Day 0) demonstrated successful burr-hole fractures in the proximal tibia (Fig. 1). Importantly, these scans also demonstrated that the burr-hole injury only passed through one side of the cortical bone and the trabecular bone leaving the other side intact (as a through and through injury demonstrates dramatically different healing kinetics). By week 1, trabecular bone had filled the burr-hole in both groups of mice (Fig. 1 arrows), and by week 4 after injury, the bone in both groups (p21^−/−^ and C57BL/6) demonstrated morphology similar to bone prior to injury (Fig. 1) and a clear injury site was not observable.

**Fig. 2:**
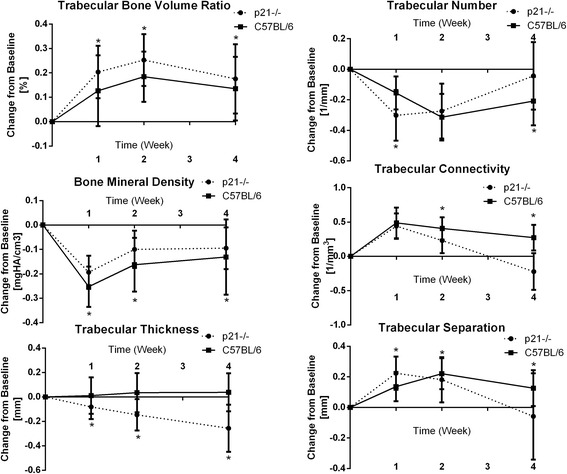
Quantitative analysis of bone histomorphometry properties obtained via μCT scanning of ROI surrounding the burr-hole fractures at day 0, week 1, 2 and 4 for C57BL/6 and p21^−/−^ mice. *p < 0.05. The error bars indicate standard deviations

To examine any differences between bone healing in C57BL/6 and p21^−/−^ mice, a number of bone parameters were quantified. All trabecular bone parameters of the ROI showed significant differences at all the time points, except trabecular connectivity at week 4 (Fig. 2). Bone morphometric data for mice are expressed after normalization based on weight (Additional file 1: Figure S1) and dimensions of the tibia (Additional file 1: Figure S2) as it was noted that p21^−/−^ has significantly varied weights and tibia dimensions. Changes in ROI morphology were observed at week 1, where p21^−/−^ showed increased bone morphological properties. In all parameters, at week 1, p21^−/−^ mice demonstrated at least twice as much change from baseline (week 0), compared to C57BL/6 mice. It was observed that even at week 4, there were significant differences in bone parameters between p21^−/−^ and C57BL/6 mice, although no difference in the healing of the injury could be visualized on the μCT images. Radiation controls for both C57BL/6 and p21^−/−^ show that radiation during μCT scanning did not influence bone quality (Additional file 1: Figure S3).

### Histological analysis of bone healing

To examine the cellular mechanism of enhanced facture repair observed in the p21^−/−^ mice, immuno-histochemical analysis was undertaken on samples collected 4 weeks post-injury. Since we have previously demonstrated that putative MSCs (Sca1, CD140a double positive cells) are increased in ‘healer’ strains of mice after cartilage injury [25], we examined if Sca1^+^, CD140a^+^ cells were also increased in the injury site in p21^−/−^ mice compared to C57BL/6 controls. At 4 weeks post injury, Sca1^+^ single positive cells were observed in both C57BL/6 and p21^−/−^ mice (Additional file 1: Figure S4 A-F) and Sca1^+^CD140a^+^ double positive cells were observed in the defect area of p21^−/−^ mice alone (Additional file 1: Figure S4 E,F). The number of positive cells was quantified, and there was no difference in the number of Sca1^+^ cells between C57BL/6 and p21^−/−^, however, while no putative MSCs (Sca1^+^CD140a^+^ double positive cells) were observed in the C57BL/6 mice, numerous MSCs were observed within the injury site of p21^−/−^ mice (Additional file 1: Figure S4 G). To determine if this observation of increased MSCs was due at an overall increase of MSCs in p21^−/−^ mice, the bone marrow was collected and assayed by flow cytometry (Additional file 1: Figure S4 H). It was observed that within the bone marrow of injured C57BL/6 vs. p21^−/−^ mice, C57BL/6 mice showed higher number of MSCs compared to p21^−/−^ mice.

### Analysis of Osteogenic and Osteoclastogenic capacity

**Fig. 3:**
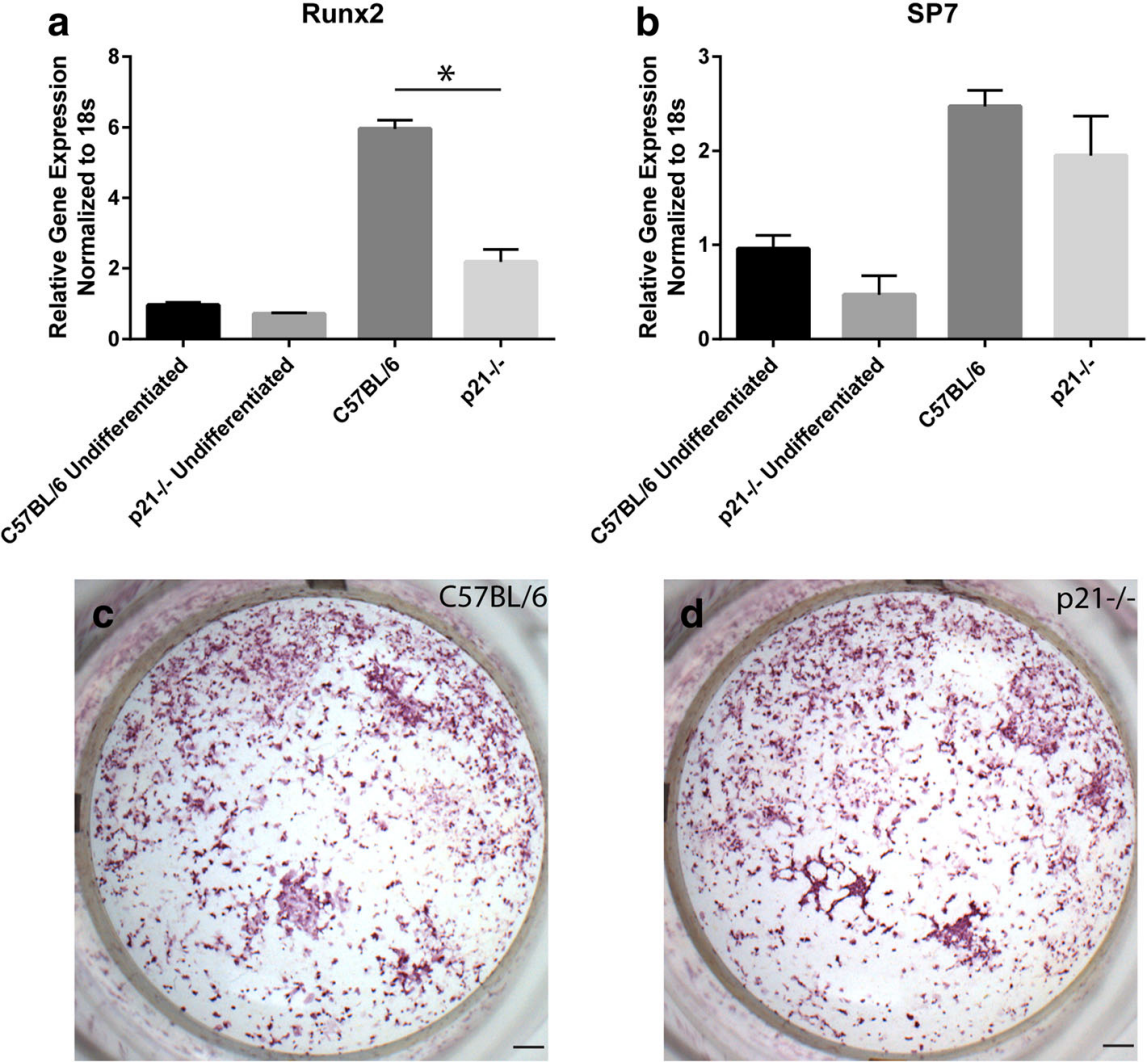
Runx2 (**a**) and sp7 (**b**) gene expression in C57BL/6 and p21^−/−^ bone marrow derived MSCs before and after osteogenic differentiation. Histological (alizarin red) staining of calcium deposits in differentiated MSCs from C57BL/6 (**c**) and p21^−/−^ (**d**) mice respectively. Error bars indicate standard deviation

Although it appeared that increased migration and/or retention of MSCs at the injury site might be responsible for the increased fracture repair observed in p21^−/−^ mice, it is still possible that mesenchymal and/or hematopoietic stem/progenitor cells in p21^−/−^ mice have greater capacity to differentiate into osteoblasts and osteoclasts respectively. To test this hypothesis, bone marrow MSCs were obtained from injured C57BL/6 and p21^−/−^ mice and induced to undergo osteogenesis (Fig. 3). While a decrease of Runx2 expression was observed in differentiated p21^−/−^ MSCs, sp7/osterix expression was similar in both p21^−/−^ and C57BL/6 mice (Fig. 3b). Furthermore, histological analysis indicates that the calcium deposits were similar in MSCs from both mice (Fig. 3c-d). Overall there was no appreciable difference in the ability of C57BL/6 or p21^−/−^ MSCs to undergo osteogenic differentiation.

**Fig. 4:**
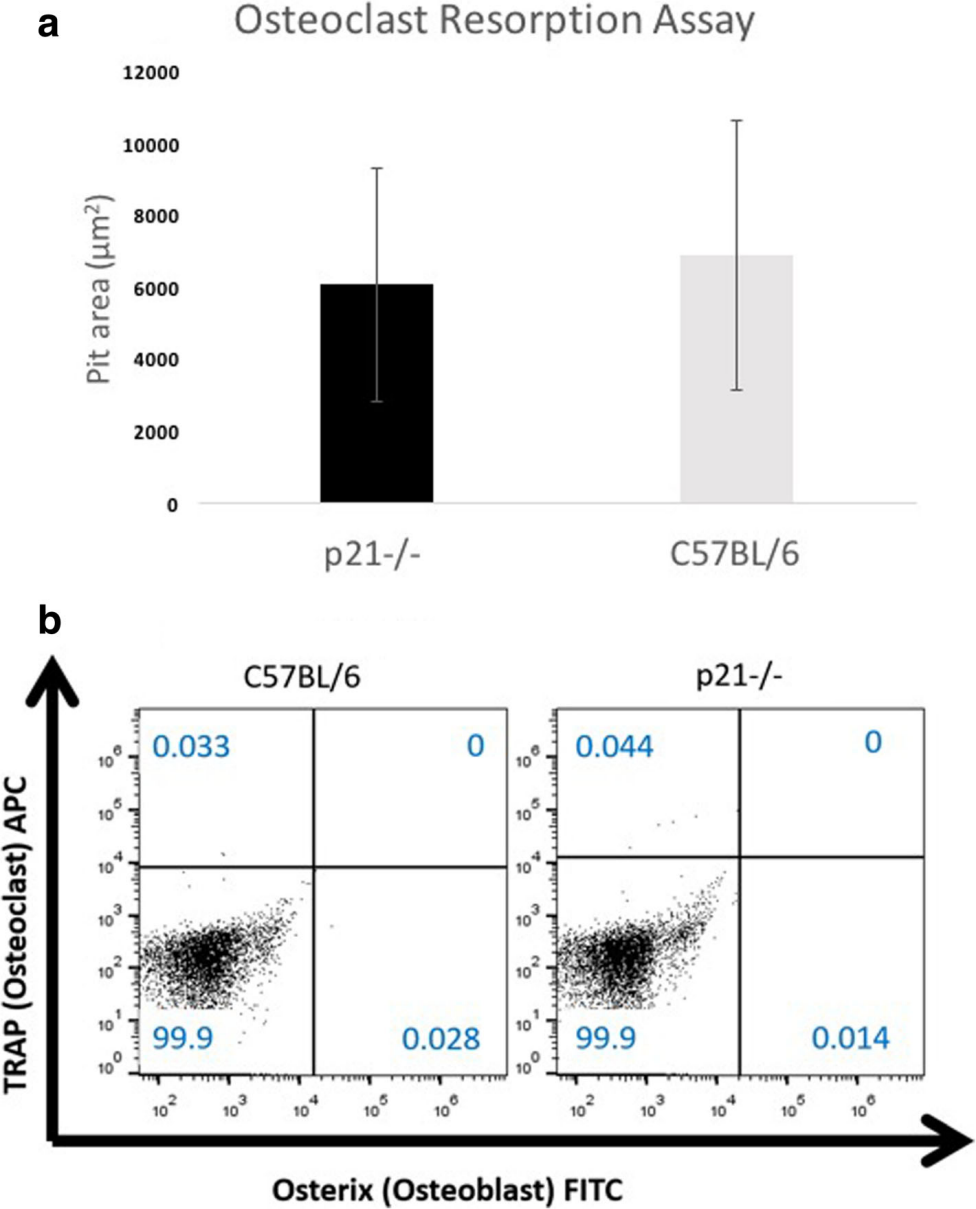
No difference was observed in the resorptive capacity of osteoclasts derived from C57BL/6 or p21^−/−^ mice (A; *n* = 4). Representative flow cytometry plots of osteoblasts and osteoclasts staining of cells isolated from the bone marrow demonstrating no difference in the number of osteoblasts or osteoclasts between C57BL/6 or p21^−/−^ mice (B; *n* = 4). Error bars indicate standard deviation

Since there was no difference observed in osteogenesis between the two mice strains, next the osteoclastogenic capacity was examined. To test this, bone marrow was isolated from C57BL/6 and p21^−/−^ mice and induced to differentiate into osteoclasts. The ability of the cells to differentiate was tested functionally through a bone resorption assay (Fig. 4, Additional file 1: Figure S5). No difference in resorptive capacity was observed between C57BL/6 and p21^−/−^ mice (Fig. 4a). Lastly, to control for there being a difference in the total number of osteoblasts and/or osteoclasts in the bone marrow of C57BL/6 or p21^−/−^ mice, the bone marrow was stained for TRAP (osteoclasts) and Osterix/sp7 (osteoblasts) (Fig. 4b). As expected, very few cells of either type were observed and no differences with observed between C57BL/6 and p21^−/−^ mice.

## Discussion

Bone is arguably one of the few tissues in the body that demonstrates true regeneration vs. repair after injury. However, there are still many clinical and pathological conditions that can negatively impact the ability of bone to recover after injury. Over the last decade there have been numerous reports of super-healer mice strains that are able to repair wounds/injuries that normally demonstrate limited repair in mammals. Specifically, both MRL and p21^−/−^ mice have demonstrated the ability to repair cartilage injuries, while most mice, including C57BL/6 cannot [7, 26, 27]. The actual mode of healing in these mice is still under debate in regards to if it is true regeneration, therefore, the purpose of this study was to determine if any enhancement in healing could be observed within super-healer mice in a tissue that normally regenerates (e.g. bone). To that end, we have shown that p21^−/−^ mice demonstrate increased facture repair compared to C57BL/6 mice in a non-critical sized defect model, and while there is no apparent difference in osteogenesis and osteoclastogenesis in p21^−/−^ vs C57BL/6 mice, increased stem cell retention at the injury site 4 weeks post injury may indicate they play a role in the enhanced healing, potentially at the initial stages during the cartilaginous callus formation. However, since earlier time points were not examined in this study, we cannot conclusively state that p21 is playing a role in the early stages of bone healing, only that p21^−/−^ mice demonstrate increased fracture repair compared to C57BL/6 controls. Additional experiments examining earlier time points would be required to test this hypothesis.

While the burr-hole model is not widely employed since it is a non-critical defect model, we employed it in this study since it was essential to examine if p21^−/−^ mice demonstrated increased repair compared to C57BL/6 and while it remains unknown if p21^−/−^ mice can repair a critical-size defect in bone, it is known that C57BL/6 mice cannot. Furthermore, the healing kinetics of the burr-hole model in the current study demonstrated reproducibility in terms of healing kinetics with previous reports using this model [28]. Using this model, it was observed that p21^−/−^ mice demonstrated enhanced bone healing, particularly at week 1, compared to C57BL/6 mice in all bone parameters measured (Fig. 2). Interestingly, we also observed small changes to the uninjured contralateral limb at week 1 (data not shown), which may indicate that changes to the trabecular bone morphology and bone mineral density may be the result of a systemic response to injury [29], however additional controlled experiments would be required to test this hypothesis. Furthermore, it should be noted that C57BL/6 and p21^−/−^ mice showed significantly different general bone morphologies, and their physical characteristics such as radial growth of the tibia also varied. Specifically, p21^−/−^ mice presented with smaller bones and overall lower total body weight, this is in contrast to previously reported work in p27^−/−^ mice that were larger than age-matched wildtype mice. p27 similar to p21 is a cyclin dependent kinase inhibitor [30], and both are from the same kinase family but vary in their C terminus and their p53 regulation, where p21 is regulated by p53 [31]. p53 regulation of p21 could potentially influence size and dimensions of bone by partially overriding effects of p21 inhibition, thereby resulting in smaller animals and could be a factor in the differences in the steady state bone morphology observed.

While previous studies in human MSCs have demonstrated that knocking down p21 leads to an increase in osteogenic potential [32], a recent study from our lab found that no increase in osteogenic differentiation potential was observed in MSCs derived from MRL super-healer mice [25]. Correspondingly, no increase in osteogenic potential was observed in this study in p21^−/−^ MSCs, suggesting that p21 may regulate osteogenesis in human MSCs, but not mouse, and also suggests that the regenerative effect observed in MRL and p21^−/−^ mice may not be the result of increased differentiation capacity of the MSCs, but instead could be an effect of increased recruitment/retention at the injury site. This hypothesis is supported by our previous study which demonstrated increased numbers of putative MSCs in the defect site of MRL mice while C57BL/6 have very few MSCs the respond to the injury [25]. In the current study, we observed a retention of MSCs even after the fracture had been healed, suggesting that these cells may also play a role in the long term remodeling of the defect.

While the contribution of other cell types in the enhanced fracture repair cannot be discounted in this study, it was clear that there was no difference in the osteoblast and/or osteoclast function (mineralization vs. resorption), and we also did not observe any change in total numbers of the cells in the bone marrow of the injury limb.

While we have been able to demonstrate that p21^−/−^ mice display increased fracture repair vs. wild-type C57BL/6 mice, this study is not without limitations. Specifically, we still do not have any direct evidence of how the injury is healing in the p21^−/−^ mice, and lineage reporter mice may be necessary to track specific cell types (including MSCs) after injury to address this question. Furthermore, since this is a constitutive knockout, we cannot rule out multiple pathways and cell types being differentially affected by the removal of p21. It is quite possible that the cartilaginous callus formed after an injury may also contribute to enhanced healing in p21^−/−^ mice as we and others have demonstrated p21 also plays a role in chondrogenesis [18]. Additionally, we cannot discount the role of inflammation in this process. After an injury to the bone, there is an immediate inflammatory response that begins the healing cascade, this inflammatory response is responsible for the recruitment of MSCs that form the initial callus bridging the injury [33]. Additional controlled experiments will been necessary to dissect out these different pathways to determine how p21 is regulating bone repair after injury and to determine if this mechanism has any potential to be translated into humans.

## Conclusions

p21^−/−^ mice show enhanced regenerative capacity compared to C57BL/6 mice, after an injury to the bone. The number of osteoblasts, osteoclasts and the osteogenic differentiation capacity of MSCs from both mice was investigated, and it is observed that these parameters did not appear to play a role in regeneration. It is therefore hypothesized that increased number of MSCs at the site of injury plays a role in regeneration, potentially owing to enhanced cartilaginous callus formation, however, this hypothesis requires future study. Overall, this study demonstrates that negative regulation of p21positively affects fracture repair, yet the mechanism by which p21 contributes to repair remains elusive.

## Additional file

Additional file 1: Figure S1. Mouse weights at each time point between age and sex matched strains. **Figure S2.** Mouse tibia dimensions in age and sex matched strains. **Figure S3.** Bone histomorphometry obtained via microCT scanning of radiation controls. **Figure S4.** Histological analysis of tibia of C57BL/6 (A) vs. p21^−/−^ (D) mice 4 weeks after injury. **Figure S5.** Representative images of pits resorbed by osteoclasts. (DOCX 5619 kb)
