# Supplementary material for: p21−/− mice exhibit enhanced bone regeneration after injury
Source: BMC Musculoskelet Disord. 2017 Nov 9;18:435. doi: 10.1186/s12891-017-1790-z (PMC5679350; doi:10.1186/s12891-017-1790-z)
Supplement: Additional file 1: Figure S1. — Mouse weights at each time point between age and sex matched strains. Figure S2. Mouse tibia dimensions in age and sex matched strains. Figure S3. Bone histomorphometry obtained via microCT scanning of radiation controls. Figure S4. Histological analysis of tibia of C57BL/6 (A) vs. p21−/− (D) mice 4 weeks after injury. Figure S5. Representative images of pits resorbed by osteoclasts. (DOCX 5619 kb) [file 12891_2017_1790_MOESM1_ESM.docx]

Supplementary Figure 1: Mouse weights at each time point between age and sex matched strains.

Supplementary Figure 2: Mouse tibia dimensions in age and sex matched strains.

Supplementary Figure 3: Bone histomorphometry obtained via microCT scanning of radiation controls for C57BL/6 and p21^-/-^ mice (n=12).


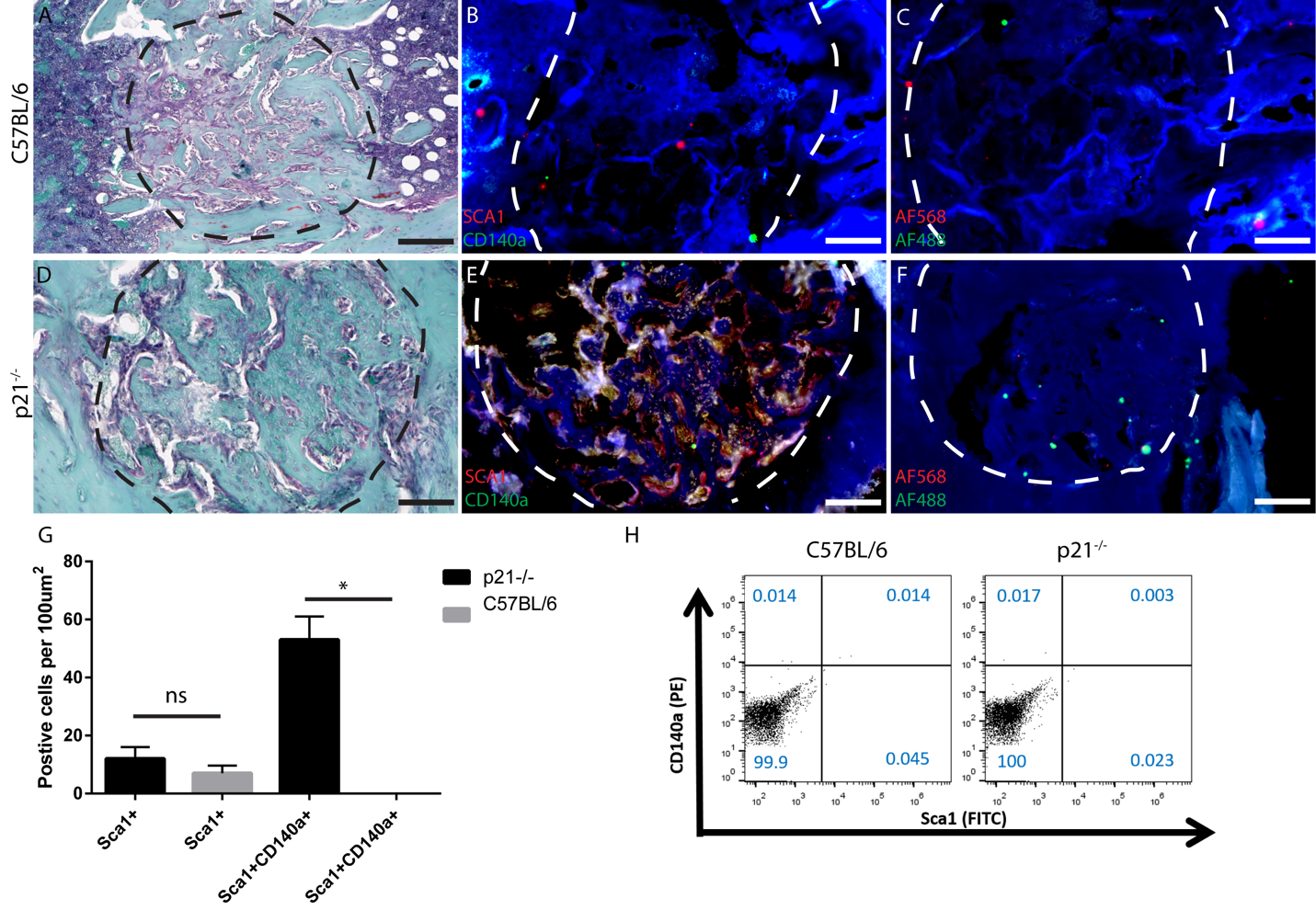


Supplementary Figure 4: Histological analysis of tibia of C57BL/6 (A) vs. p21^-/-^ (D) mice 4 weeks after injury. Presence of Sca1 and CD140a expression cells suggests undifferentiated mesenchymal stem cell status (B,E). Secondary controls show limited non-specific staining (C,F). Quantitative analysis of Sca1 and CD140a positive cells demonstrates increased double positive cells in p21^-/-^ mice 4 weeks after injury (G). Flow cytometry plots illustrating cells that are positive for Sca1 and CD140a exclusively as well as cells positive for both these markers (H). p*<0.05.


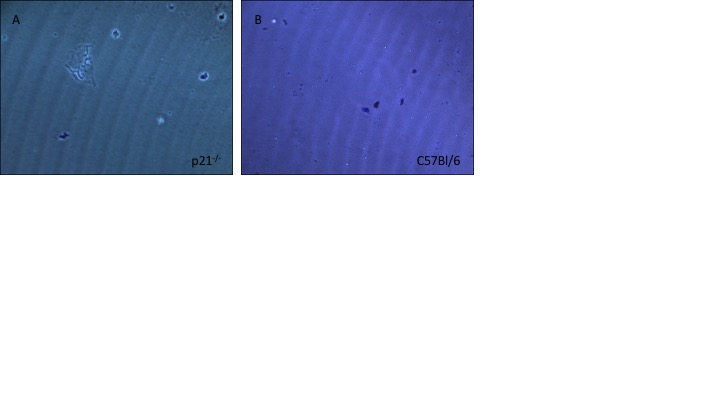


Supplementary Figure 5: Representative images of pits resorbed by osteoclasts in (A) p21^-/-^ and (B) C57BL/6 mice.
